# *Paramphistomum daubneyi*: characteristics of infection in three lymnaeid species

**DOI:** 10.1051/parasite/2012194445

**Published:** 2012-11-15

**Authors:** R. Sanabria, A. Titi, A. Mekroud, P. Vignoles, G. Dreyfuss, D. Rondelaud, J. Romero

**Affiliations:** 1 CEDIVE, Fac. Cs. Veterinarias, Universidad Nacional de La Plata Alvear 803 (7130) Chascomús, Buenos Aires Argentina; 2 PADESCA Laboratory, Department of Veterinary Sciences, University Mentouri Constantine 25100 El Khroub Algeria; 3 INSERM U 1094, Faculties of Medicine and Pharmacy 87025 Limoges France

**Keywords:** *Galba truncatula*, *Lymnaea neotropica*, *Lymnaea viatrix*, *Paramphistomum daubneyi*, experimental infection, metacercaria, redia, *Galba truncatula*, *Lymnaea neotropica*, *Lymnaea viatrix*, *Paramphistomum daubneyi*, infestation expérimentale, métacercaire, rédie

## Abstract

Experimental infections of two South American lymnaeids (*Lymnaea neotropica* and *L. viatrix* var. *ventricosa*) with *Paramphistomum daubneyi* were carried out to determine if these snail species could sustain larval development of this digenean and, if so, to specify their potential for cercarial production. A French population of *Galba truncatula* infected and raised according to the same protocol served as controls. In both experiments, prevalence of *P. daubneyi* infections in snails did not significantly differ from each other. In snail groups evaluated for cercarial shedding (first experiment), a significantly lower number of shed cercariae was noted for *L. neotropica*, while those from *G. truncatula* and *L. v. ventricosa* did not differ significantly from each other. Dissection of infected snails at day 65 post-exposure at 20 °C (second experiment) found significantly lower burdens of *P. daubneyi* rediae and cercariae in the bodies of *L. neotropica* than in those of *G. truncatula* and *L. v. ventricosa*. Compared to total cercarial production observed in dissected snails, the percentage of cercariae which exited from snails was 75.6% for *G. truncatula*, 21.6% for *L. neotropica*, and 91.4% for *L. v. ventricosa*. This last species seems to be a good candidate for metacercarial production of *P. daubneyi*.

## Abstract (fr)

Des infestations expérimentales de deux limnées sud-américaines (*Lymnaea neotropica* et *L. viatrix* var. *ventricosa*) avec *Paramphistomum daubneyi* ont été réalisées pour déterminer si ces espèces peuvent assurer le développement larvaire de ce Digène et, dans l’affirmative, pour préciser leur potentiel dans la production cercarienne. Les témoins sont constitués par une population française de *Galba truncatula* infestée et élevée selon le même protocole. Dans les deux expériences, les prévalences de l’infestation par *P. daubneyi* chez les mollusques sont proches les unes des autres. Chez les groupes suivis pour les émissions cercariennes (première expérience), le nombre de cercaires émises est significativement plus faible chez *L. neotropica*, tandis que les chiffres notés chez *G. truncatula* et *L. v. ventricosa* ne présentent pas de différence significative. Si les mollusques infestés sont disséqués au 65ème jour post-exposition à 20 °C (seconde expérience), les charges rédienne et cercarienne trouvées dans le corps des *L. neotropica* sont significativement plus faibles que celles notées chez *G. truncatula* et *L. v. ventricosa*. Par rapport à la production cercarienne totale chez les mollusques disséqués, le pourcentage de cercaires qui sont émises est de 75,6 % chez *G. truncatula*, 21,6 % chez *L. neotropica* et de 91,4 % chez *L. v. ventricosa*. Cette dernière espèce semble être un bon candidat pour la production métacercarienne de *P. daubneyi*.

Parasitosis due to *Paramphistomum daubneyi* (rumen fluke) affects ruminants in numerous countries of Europe (see the review by Sey, 1980), Middle East (Ozdal *et al*., 2010), Africa (Dinnik, 1962; Pacenowsky *et al*., 1987; Seck *et al*., 2008), India and New Zealand (Sey, 1980). Even though this digenean is mainly found in cattle (Dorchies *et al*., 1998, Dorchies *et al*., 2000), natural and experimental infections can develop in other ruminants such as sheep (Vignoles *et al*., 2008) and goats (Silvestre *et al.*, 2000). In Africa and Europe, the main snail host is *Galba truncatula* (Sey, 1979; Brown, 1994). However, other European lymnaeids such as *Omphiscola glabra*, *Stagnicola fuscus* and *S. palustris* (Abrous *et al*., 1998; Degueurce *et al*., 1999) may support larval development of *P. daubneyi* if they have been co-infected with another digenean, *Fasciola hepatica*. Under these conditions, snails may harbour larval forms of the first parasite, the second, or both (Rondelaud *et al*., 2009).

The aim of the present paper was to identify lymnaeids other than European species, which may act as intermediate hosts in the life cycle of *P. daubneyi*. Due to the global distribution of this digenean (see above), we chose amphibious lymnaeids from South America for the following two reasons: *i*) numerous cattle and other animals were introduced to this continent in the 16th century by Spanish and Portuguese (Mas-Coma *et al*., 2009); and *ii*) the paramphistomid currently present in cattle from Spain and Portugal is *P. daubneyi* (Diaz *et al*., 2007; Arias *et al*., 2011). Experimental infections of two South American lymnaeids: *Lymnaea neotropica* and *L. viatrix var. ventricosa* with a French isolate of *P. daubneyi* miracidia were thus carried out to determine their capacity to sustain the development of larval forms and to specify the quantity of *P. daubneyi* cercariae these snail species might produce when the method proposed by Rondelaud *et al*. (2007) for amphibious snails was used for their breeding. Controls came from a French population of *G. truncatula* infected and raised according to the same protocol.

## Material and Methods

The two South American lymnaeids have been raised under laboratory conditions at CEDIVE (Faculty of Veterinary Sciences, National University of La Plata, Buenos Aires, Argentina) since 2008 (*L. neotropica*) and 1996 (*L. v. ventricosa*). The first species came from a population living in San Pedro, Buenos Aires, Argentina (33° 40’ S, 59° 39’ W), while *L. v. ventricosa* originated from Paysandú, Uruguay (36° 00’ S, 57° 30’ W). As the systematics of South American lymnaeids is controversial and cannot be done with the use of single morphological criteria (Duffy *et al*., 2009; Mera y Sierra *et al*., 2009), the identification of these two species was performed using PCR-RFLP and sequencing of the ITS-1 segment of their nuclear rDNA (Sanabria *et al*., 2012). The French population of *G. truncatula* was collected from a road ditch (45° 55’ 33’’ N, 2° 2’ 33’’ E) in the commune of Saint-Michel-de-Veisse, department of Creuse. The habitat of this last population was located on siliceous soil so that the upper shell height of adults (8–9 mm) was the same as that of South American adults. To obtain *P. daubneyi* eggs, adult worms were collected from the rumen of infected cattle at the slaughterhouse of Limoges (France) and dipped in a physiological saline (ClNa, 0.9 %; glucose, 0.45 %) solution before being placed at 37 °C for three hours. Eggs were washed several times with spring water and incubated at 20 °C in the dark according to the report by Ollerenshaw (1971) for *F. hepatica*.

Two experiments were carried out in the present study. The aptitude of each snail species as an intermediate host of *P. daubneyi* was studied in experiment (A). One hundred snails, each measuring 4 mm in height, were randomly chosen from each population and subjected to individual quadrimiracidial exposures for four hours at 20 °C. The choice of four miracidia per snail came from the results of a preliminary experiment with these three populations and routine bimiracidial infections. Under these conditions, 3.2 % and 6.1 % of *L. neotropica* and *L. v. ventricosa*, respectively, were infected (instead of 91.8 % of *G. truncatula*) but most snails died without cercarial shedding (unpublished data). Snails were then raised in groups of ten individuals in 14 cm Petri dishes during the first 30 days according to the method of Rondelaud *et al*. (2007). They were maintained on dried lettuce leaves and dead grass leaves (*Molinia caerulea*), while several stems of live *Fontinalis* sp. ensured oxygenation of the water layer. Petri dishes were placed at a constant temperature of 20 °C (± 1 °C), with a natural photoperiod of ten hours light. At day 30 post exposure (p.e.), each surviving snail was placed in a 35 mm Petri dish with pieces of dead grass, lettuce and spring moss, and also kept at 20 °C. Snails were observed daily to change spring water and food if necessary, and to count metacercariae before their removal from dishes. When the first cercarial shedding occurred, the surviving snails were subjected to a thermal shock every three days by placing their Petri dishes at 10–13 °C for three hours (outdoors) to stimulate cercariae exit. Experiment (B) was carried out on order to determine cercarial production of *P. daubneyi* in snails dissected at day 65 p.e. at 20 °C (the first cercarial shedding generally occurred at the end of week 9 p.e.). Each experimental group contained 50 snails. Snail exposure to miracidia and their breeding were similar to those of experiment (A). At day 65, each snail was dissected under a stereomicroscope to count free white procercariae and yellowish brown cercariae of *P. daubneyi*. Free rediae were also taken into account.

The first two parameters were snail survival at day 30 p.e. and prevalence of *P. daubneyi* infection (calculated in relation to the number of surviving snails at day 30 p.e.). Prevalence took into account the number of cercariae-shedding snails (CS snails) and infected individuals, which died without cercariae exit (NCS snails) in experiment (A), and all snails carrying larval forms of *P. daubneyi* in experiment (B). For each parameter, the difference between the values noted for the three snail groups was analyzed using a χ2 test. In experiment (A), the growth of CS snails during the experiment, length of the prepatent period, length of the patent period, and total number of metacercariae were also taken into account. In experiment (B), the number of free rediae, quantity of procercariae and that of free cercariae were considered. Individual values recorded for these last seven parameters were averaged and their standard deviations calculated considering snail groups. One-way analysis of variance was used to establish levels of significance. All statistics were made using Statview 5.0 software.

## Results and Discussion

Compared to the survival of control *G. truncatula* at day 30 p.e. (Table I), the rate of *L. neotropica* was significantly greater while that of *L. v. ventricosa* was lower (χ2 = 11.50, *P* < 0.01). The differences between prevalence of infection, growth of CS snails, lengths of prepatent periods, and those of patent periods were insignificant. In contrast, the number of metacercariae shed by *L. neotropica* was significantly lower (*F* = 4.70, *P* < 0.05) than those released by the other two lymnaeids. Contrary to *G. truncatula*, which shed its cercariae during several waves, most *L. neotropica* and *L. v. ventricosa* released these larvae during a single shedding wave and died after (data not shown).

**Table I.** Main characteristics of *P. daubneyi* infection in three species of lymnaeid snails (experiment A).

Parameters	*G. truncatula*	*L. neotropica*	*L. v. ventricosa*
Number of preadult snails at exposure	100	100	100
Number of surviving snails at day 30 p.e. (survival rate %)	68 (68.0)	81 (81.0)	59 (59.0)
Number of CS snails	25	34	16
Number of NCS snails	11	7	7
Prevalence of infection (%)	52.9	50.6	54.5
Growth of CS snails during the experiment (mm)*	2.7 (0.6)	2.6 (0.8)	2.7 (0.7)
Length (days)*:			
– prepatent period	69.3 (3.1)	73.1 (4.6)	71.5 (2.9)
– patent period	24.8 (1.9)	27.6 (6.0)	25.0 (8.5)
Total number of metacercariae*	169.2 (76.1)	37.4 (25.2)	253.0 (151.2)

* Mean value (S.D.); CS snails, cercariae-shedding snails; NCS, infected snails without cercarial shedding

Table II gives the results of experiment (B). Significant differences (χ2 = 18.05, *P* < 0.001) were noted between the survival of *G. truncatula* at day 30 p.e. and the rates of the other two lymnaeids. As for experiment (A), prevalence of *P. daubneyi* infection in the three snail groups did not differ significantly from each other. Significantly lower numbers of free rediae (*F* = 4.26, *P* < 0.05) and free cercariae (*F* = 3.58, *P* < 0.05) were seen for *L. neotropica*, whereas the differences existing between the other two lymnaeids were insignificant. In all three snail groups, the numbers of free procercariae were similar and no significant difference was noted.

**Table II.** Counts of *P. daubneyi* rediae and cercariae in three species of lymnaeids at day 65 p.e. at 20 °C (experiment B).

Parameters	*G. truncatula*	*L. neotropica*	*L. v. ventricosa*
Number of preadult snails at exposure	50	50	50
Number of surviving snails at day 30 p.e. (survival rate %)	37 (74.0)	45 (90.0)	26 (52.0)
Number of snails containing cercariae (prevalence %)	19 (51.3)	22 (48.8)	13 (50.0)
Number of free rediae*	34.4 (5.1)	26.2 (8.6)	39.5 (6.2)
Number of free procercariae*	81.4 (21.0)	62.5 (15.6)	99.5 (29.9)
Number of free cercariae*	142.4 (32.3)	110.2 (27.4)	177.3 (52.9)
Total cercarial production*	223.8 (27.3)	172.7 (21.8)	276.8 (37.1)

* Mean value (S.D.).

If the number of shed cercariae (Table I) was compared to total cercarial production within the snail body (Table II), the percentage of larvae which exited from the snail was 75.6 % for *G. truncatula* and 91.4 % for *L. v. ventricosa*, while it was only 21.6 % for *L. neotropica*.

As the intermediate hosts of *P. daubneyi* in Africa and Europe are *G. truncatula* and *Lymnaea peregra* (Sey, 1979), the findings reported in the present study demonstrated that *L. neotropica* and *L. v. ventricosa* also were potential snail hosts for this digenean. Two perhaps complementary hypotheses may be proposed to explain these results. First, *P. daubneyi* is apparently present in South America, at least in Argentina and Uruguay, even though other paramphistomids such as *Paramphistomum cervi* (Nascimento *et al*., 2006) and *Paramphistomum leydeni* (Sanabria *et al*., 2011a, b) have already been reported on this continent. Secondly, larval development of *P. daubneyi* might occur in several species of the genus *Galba/Fossaria*. An argument supporting this last approach was the report by Castro-Trejo *et al*. (1990) with *P. cervi* larvae developing in three lymnaeids (*Lymnaea cubensis, L. humilis* and *L. palustris*).

In the two South American lymnaeids, there was a strong disparity in snail response to larval development of *P. daubneyi*. Compared to control *G. truncatula*, the mean redial and cercarial burdens in *L. v. ventricosa* (Table II) were slightly higher and this snail population shed 91.4 % of its cercariae. In contrast, in *L. neotropica*, redial and cercarial burdens were significantly lower (Table II) and only 21.6 % of cercariae were shed. These findings may be interpreted as consequences of a still incomplete adaptation between both partners and this incomplete adaptation would be greater for *L. neotropica* than for *L. v. ventricosa*. This hypothesis is supported by the fact that numerous South American lymnaeids shed their cercariae during a single wave. According to Rondelaud *et al*. (2009), the exit of *F. hepatica* cercariae during a single wave of one or several days, followed by snail death, indicated an incomplete adaptation between snail population and the parasite.

Low lengths of patent periods were noted for infected *L. neotropica* and *L. v. ventricosa* in the present study. In contrast, longer patent periods up to a mean of 96.3 days for *L. v. ventricosa*, for example, infected by *Fascioloides magna* (Sanabria *et al*., 2012) were reported. As control *G. truncatula* also showed low lengths of patent periods (Table I), these findings can be explained by the use of four miracidia per snail.

## Conclusion

Even if the miracidial burden of *P. daubneyi* used in the present study seemed to clearly reduce life expectation of infected *L. v. ventricosa*, this species is a good candidate for metacercarial production of this digenean. As breeding of this snail in the laboratory using the method by Rondelaud *et al*. (2007) was easier than for *G. truncatula*, this lymnaeid might replace *G. truncatula* as a snail host for *P. daubneyi*. However, a study on the viability of metacercariae produced by *L. v. ventricosa* and their development into adults in the definitive host is still necessary to optimize the characterization of this snail species.
